# Commentary: Attentional tradeoffs maintain the tracking of moving objects across saccades

**DOI:** 10.3389/fnsys.2015.00188

**Published:** 2016-01-11

**Authors:** Kieran Gallagher

**Affiliations:** Department of Neurobiology, Harvard UniversityCambridge, MA, USA

**Keywords:** saccade, tracking, remapping, attention, dynamic displays

We constantly move our eyes, yet our perception of the world is stable due to compensatory neuronal mechanisms that occur around the time of saccades. One proposed distortion is “attentional remapping,” where the point of attention is shifted to the post-saccadic target before the eyes begin to move. Attentional remapping has been observed during saccade preparation in a static display (Rolfs et al., 2011). This has been seen neuronally as well; before a saccade, some parietal neurons transiently shift their receptive field to react to stimuli that will be in the receptive field after the saccade (Duhamel et al., 1992).

Szinte et al. (2015) focus on how attention shifts around the time of saccades during attentive tracking in a dynamic display. The authors confirm that attentional tracking continuously shifts the focus of attention along the object's predicted path. They show that, when preparing to make an eye movement, subjects shift attention to the retinal position the object is expected to occupy after the saccade. Thus, the focus of attention moves before the eyes move to the new point of fixation.

In the task, subjects attentively track an object in a clockwise direction, while fixating on a target in the center (Figure 1A). During the trial, a motion pulse occurs in one of six locations, moving either up, down, left or right (Figure 1B). In the fixation task, subjects indicate the direction of the motion pulse at the end of the trial. In the saccade task, a target appears during the trial, and subjects move their eyes to this target (Figure 1C). A key part of the task is that the circles opposite to the tracked location lie in the location where attention is remapped as a result of the saccade. Importantly, the pulse occurs during saccade preparation, so subjects' ability to detect the motion pulse at specific locations reflects where attention is allocated immediately before making an eye movement.

**Figure 1:**
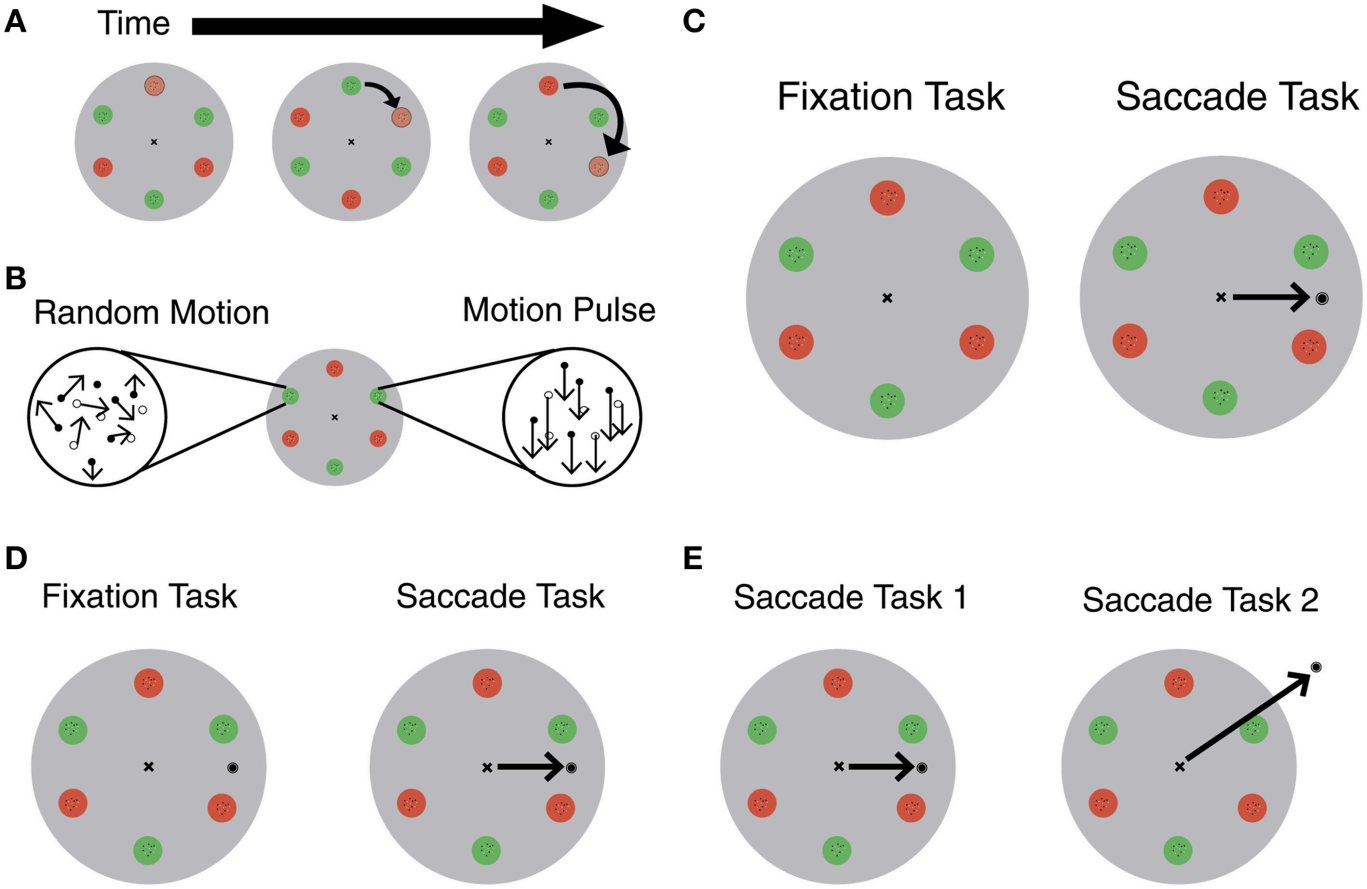
**(A)** Individual red and green circles change color at regular intervals to create circular apparent motion. Subjects are cued to start at one circle and track in a clockwise direction around the six stimulus location. Adapted from Szinte et al. (2015). **(B)** Random dot kinematograms (RDKs) were positioned inside each red and green circle. One RDK undergoes a motion pulse at some point during the trial. Adapted from Szinte et al. (2015). **(C)** Szinte et al. (2015) task: subjects fixated, and covertly tracked a “moving” stimulus until there was a change in one of the RDK's motion. Subjects indicate the direction of the motion pulse on the keyboard (fixation task). In the saccade task, a target appeared randomly during the trial and subjects saccade to the target (black arrow in saccade task). Adapted from Szinte et al. (2015). **(D)** Alternative task to control for saccade target onset: target appears on screen during fixation and saccade trials. In the saccade task, subjects saccade in response to an unpredictable auditory cue (black arrow in saccade task). **(E)** Alternative task to isolate the effect of attentional remapping: compare original saccade task with a task that includes an irrelevant saccade to isolate the effect of attentional remapping (black arrows).

The authors used the subjects' ability to correctly report the direction of the motion pulse as evidence of when and where attention was focused. They show that performance was best when the motion pulse occurred at the tracked location in the fixation task and worst at the opposite location.

Performance decreased at the tracked location prior to saccade onset, and the authors interpreted this as support for attentional remapping. The decrease in performance at the tracked location could have occured because the appearance of a new visual stimulus “distracted” subjects via exogenous attention. Given the experimental setup and the established history of exogenous attention (Bisley and Goldberg, 2010), it may be more parsimonious to attribute the decrease in performance that occurred at these locations to attentional remapping.

Szinte et al. (2015) try to address this concern by measuring performance relative to the time of saccade target onset. They are looking to see if performance falls immediately after saccade target onset and if performance recovers later when the saccade target no longer holds the subjects' transient attention. These results would show that saccade target onset rather than attentional remapping likely causes the drop in attention in the saccade task. Performance fell immediately after saccade onset. However, performance was decreased at the tracked location for 350 ms following saccade target onset. It should have recovered after 100 ms if the appearance of the saccade target were the main cause for this decreased performance, as this is the time that the saccade target would no longer hold the subjects' transient attention (Nakayama and Mackeben, 1989).

This result is consistent with attentional remapping but the influence of the appearance of the saccade target on attention is unclear. It is impossible to distinguish the effect of remapping from the effect of saccade target onset. This momentary distractor may have affected the timescale or magnitude of the attentional modulation attributed to the saccade.

The authors could have avoided this confound by showing the saccade target on the screen throughout the duration of the trial. (Figure 1D). In saccade trials, they could instruct participants to make a saccade in response to an unpredictable auditory cue. The only difference between the saccade and fixation trials in this set-up is the act of making the eye movement.

Furthermore, the task does not isolate the effect of attentional remapping from other effects of making an eye movement. The authors argue that the decrease in performance at the tracked location occurs because the subjects are shifting attention to the opposite visual field. However, it is also possible that performance worsens at the tracked location because of another feature of making an eye movement such as inherent physiological delays in sending copies of the motor command signals corollary discharge from the midbrain to the cerebral cortex (Sommer and Wurtz, 2002). Such delays may be different for each visual hemifield because of cross-hemisphere relays.

This subtle distinction could be explored in future experiments. For example, researchers could use this same experimental set-up to compare the difference in performance between two saccade tasks (Figure 1E). The first task would be identical to the saccade task from this experiment. In the second task, the saccade target would be in a completely new and irrelevant location, such as the top of the screen. In these two tasks, the only difference is where the subjects remap attention. The first task involves remapping attention between regions that are relevant to the task. The second task involves remapping attention away from relevant targets. The second task provides a baseline and makes it possible to isolate the effect of attentional remapping, if any.

On the other hand, future neurophysiological work could benefit from the behavioral paradigm introduced by Szinte et al. (2015). Previous studies have found that the lateral intraparietal area (LIP) and the frontal eye field (FEF) are involved in attentive tracking as well as predicting the location of an object after a saccade (Moore, 2006; Mayo and Sommer, 2010). The authors suggest that FEF and LIP index the anticipated location of an object and alter processing in the primary visual cortex accordingly. Neuronal recordings, or even reversible inactivation using a comparable task could delineate LIP's involvement in attentional remapping. Such results would help solidify and expand on the case for attentional remapping made by Szinte et al. (2015).

## Conflict of interest statement

The author declares that the research was conducted in the absence of any commercial or financial relationships that could be construed as a potential conflict of interest.
